# Supervised Health Stage Prediction Using Convolutional Neural Networks for Bearing Wear

**DOI:** 10.3390/s20205846

**Published:** 2020-10-16

**Authors:** Sungho Suh, Joel Jang, Seungjae Won, Mayank Shekhar Jha, Yong Oh Lee

**Affiliations:** 1 Smart Convergence Group, Korea Institute of Science and Technology Europe Forschungsgesellschaft mbH, 66123 Saarbruecken, Germany; s.suh@kist-europe.de (S.S.); wkddydpf@korea.ac.kr (J.J.); 2015100729@khu.ac.kr (S.W.); 2 Department of Computer Science, TU Kaiserslautern, 67663 Kaiserslautern, Germany; 3 Department of Computer Science and Engineering, Korea University, Seoul 02841, Korea; 4 Department of Software Convergence, Kyung Hee University, Yongin-si 17104, Korea; 5 Centre de Recherche en Automatique de Nancy (CRAN), UMR 7039, CNRS, University of Lorraine, 54506 Vandoeuvre CEDEX, France; mayank-shekhar.jha@univ-lorraine.fr

**Keywords:** fault detection, convolutional neural networks, feature extraction, machinery prognostics, health stage division

## Abstract

Early detection of faults in rotating machinery systems is crucial in preventing system failure, increasing safety, and reducing maintenance costs. Current methods of fault detection suffer from the lack of efficient feature extraction method, the need for designating a threshold producing minimal false alarm rates, and the need for expert domain knowledge, which is costly. In this paper, we propose a novel data-driven health division method based on convolutional neural networks using a graphical representation of time series data, called a nested scatter plot. The proposed method trains the model with a small amount of labeled data and does not require a threshold value to predict the health state of rotary machines. Notwithstanding the lack of datasets that show the ground truth of health stages, our experiments with two open datasets of run-to-failure bearing demonstrated that our method is able to detect the early symptoms of bearing wear earlier and more efficiently than other threshold-based health indicator methods.

## 1. Introduction

Rotary machinery is extensively used in modern industry for civilian and military applications such as compressors, turbines, aircraft engines, etc. High service loads and varying operational conditions lead to the fault/failure of the machinery via the degradation of one or more critical components. The detection of fault/impending failure is very significant in avoiding catastrophic accidents and ensuring the safe operation of the machinery [1].

**Figure 1:**
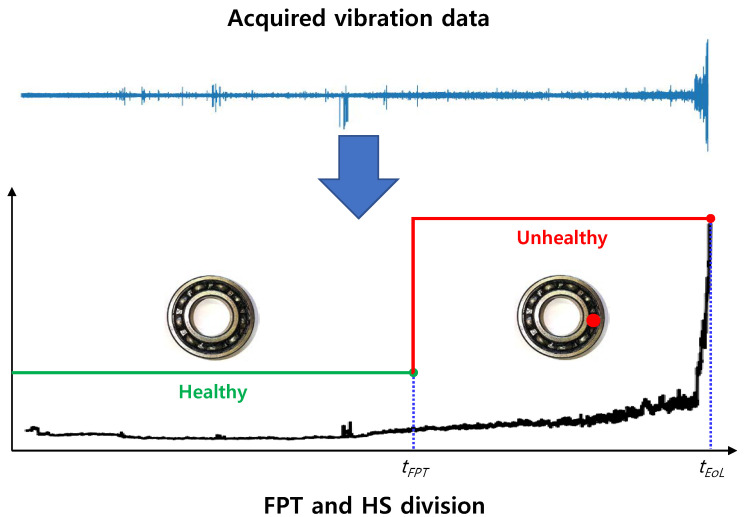
Degradation process with first predicting time (FPT) and health stage (HS) division.

Early fault detection is significantly influenced by the ability of any method to predict the health stage (HS) of the machinery. To that end, the HS division procedure divides the continuous degradation process into two or several HSs according to the varying trends of the health indicator (HI) [2]. HS division is different from fault detection or fault diagnosis in that, while the latter aims to detect the appearance and severity of the fault, the former aims to facilitate the remaining useful life (RUL) prediction methods by dividing the degradation progression into two or several stages. Typically, in the two-stage HS division, a distinction between healthy (nominal functioning) and unhealthy (anomalous) states is made to trigger the RUL prediction process. As such, the start time of the unhealthy stage is known as the first predicting time (FPT). As shown in Figure 1, the continuous degradation process is divided into two HSs by determining FPT.

Traditional methods of HS division have involved the intervention of experts or qualified operators, thereby remaining subjective to operator’s perception and sensitivity, and the ever-changing operational conditions. For more reliability, methods of testing the components systematically have been proposed. Most methods utilize HI and the HS division is made based on a prescribed threshold defined a priori [3]. The root mean square (RMS) is widely used to represent HIs and is further employed to identify the initial point of degradation when the RMS exceeds a pre-specified threshold [4]. On the other hand, unsupervised approaches aim to extract those features in an unsupervised manner that accurately represents the HI, thereby avoiding the need to identify a suitable representation of HI. For example, the auto-encoder (AE)-based method avoids the identification of HI by extracting useful features of faults in an unsupervised manner [5,6]. However, AE for HS division still possesses the same challenge: assigning an appropriate threshold that will detect the formation of a fault early enough for diagnosis, while, at the same time, leading to minimal false alarms.

Moreover, this threshold should be adaptable to time-varying operational conditions and environments. If the threshold is set low for early FPT, the false alarm rate goes up and the maintenance system becomes highly inefficient. If the threshold is set too high, the fault detection is not made early enough for precautionary measures. Previous methods also lack appropriate feature extraction methods for detecting faulty features in the early stage of fault development. In summary, existing methods of HS division rely heavily upon the choice of efficient HI (such as RMS) and pre-specified thresholds. Although unsupervised methods (such as AE) promise the extraction of efficient HIs, the problem of the pre-selection of thresholds persists. The paradox of determining an appropriate threshold and the lack of an efficient feature extraction method has demonstrated the need for an efficient early fault detection mechanism.

In this work, we propose a supervised data-driven method for HS division, that avoids usage of a pre-specified HI as well as the employment of pre-specified thresholds. The principle idea presented in this paper involves supervised training of a binary regression model using the early stage (nominal functioning) samples and last stage (near to complete failure) samples from a run-to-failure bearing degradation database. Unlike conventional HI methods, there is no need to set the threshold and it only needs a small number of labels. The laborious task of labeling, which is typically associated with supervised methods, is also avoided as a simple distinction is made between the early and last stages of component degradation containing relatively smaller sets of data to be labeled. The proposed method has two phases. Phase one consists of transforming the time series data of raw vibration data into a nested-scatter plot image (NSP) for feature extraction. In phase two, these images are used to train a binary regression model using convolutional neural networks (CNN), which are tested on degradation datasets provided by the center of Intelligent Maintenance Systems (IMS), the University of Cincinnati [7], and the Fanche-Comte Electronics Mechanics Thermal Science and Optics—Sciences and Technologies Institute (FEMTO) [8,9]. The superiority of the proposed method is verified through a comparison with previous detection methods in terms of FPT: RMS in the signal-based method and AE in the unsupervised data-driven method. In addition, the features extracted by CNN are analyzed to verify the reliability of the predicted FPT [10,11].

The contributions of this paper can be summarized as follows.

- A CNN-based binary regression model combined with NSP rids the necessity of designating a threshold a priori since the binary regression model accomplishes that task. Instead, a simple trigger mechanism is utilized in the binary regression model output to distinguish healthy and unhealthy states. Even though supervised learning is proposed, the inherent labeling procedure is simplistic and the training process requires a comparatively very small amount of labeled data.
- The proposed method detects degradation patterns of rotary machine elements earlier than the commonly used RMS-based approaches, or AE-based methods. The feature analysis shows that the proposed method is able to extract HS features earlier and more distinguishably than previous threshold-based HS division methods.

This paper is organized as follows. Section 2 briefly introduces the related works to predict the HS. Section 3 describes the details of the proposed algorithm, and Section 4 presents the experimental results. Section 5 contains the discussion and Section 6 concludes the paper.

## 2. Related Works

To improve the accuracy of the HS prediction process, the methods of systematically testing the machine condition were proposed. These methods are categorized as model-based, signal-based, and data-driven methods [12].

Model-based methods monitor the health state of rotary machines by using mathematical models that utilize the physical relationships between the control to the motor and its dynamic response in the time or frequency domain [13]. Rotary machines often involve complex non-linear relationships among the system variables and operational conditions which are rarely captured by mathematical models accurately.

Signal-based methods use time domain, frequency domain, or time–frequency feature analysis to estimate HSs from measured signals [14]. This approach often requires a priori knowledge of various impending faults (fault signatures) which may vary depending upon various operational conditions, calling for manual fine-tuning or calibration of thresholds employed for HS detection. The root mean squared (RMS) value, one of the represented HI, is used for identifying the initial point of degradation when this RMS value exceeds a pre-specified threshold [4]. More advanced HIs have been proposed such as the Mahalanobis distance [15] and the Chebyshev inequality function [16]. Recently, many threshold-based HS division works employing statistically derived HIs have been proposed. For example, [17] used adaptive thresholds for the integrated detection of FPT and prognostics. As mentioned, HI computed by the signal-based method has a limitation in relation to manual threshold value setting that could vary based on the operation conditions.

The data-driven method applies machine learning techniques to extract characteristics from a machine directly from the measured data. Recently, deep learning and pattern recognition have demonstrated extensive utility in this domain. The data-driven method can be further categorized into supervised [18] and unsupervised methods [19] in which supervised methods require training sets that are labeled. The labeling of the dataset is a time-consuming process prone to human error. The unsupervised method, on the other hand, does not require manual labeling but tries to detect anomalies that deviate from the nominal behavior.

Belmiloud et al. [20] used wavelet packet decomposition to extract features as the model input and proposed a deep CNN-based method to construct HI. Guo et al. [21] proposed a CNN-based HI construction method with little prior knowledge to extract features. She et al. [22] proposed a multi-channel CNN with an exponentially decaying learning rate to construct the HI with the original multi-channel raw vibration signals. Li et al. [23] proposed an RUL prediction method based on multi-scale feature extraction using CNN. Before predicting RUL, they determined the FPT by using kurtosis and used short-time Fourier transform to process raw vibration signals and obtain the time–frequency domain information. Then, the CNN with multi-scale feature extraction predicted the RUL. However, such methods still possess the same challenge: assigning an appropriate threshold.

## 3. Supervised Health Stage Prediction

### 3.1. Image Transformation of Vibration Signals

NSP is a data wrangling method that transforms correlated time series data into an image for multi-variate correlation analysis [24]. It is a matrix representation similar to the heat map of the quantized value of time series data. Each vibration signal is quantized and mapped into nested clusters. The cumulative number of signal values in the nested cluster is normalized in order to represent the density of the nested cluster. Although NSP removes the non-stationarity of the time sequence, it is an efficient imaging method for multi-variable correlation analysis [24,25].

**Figure 2:**
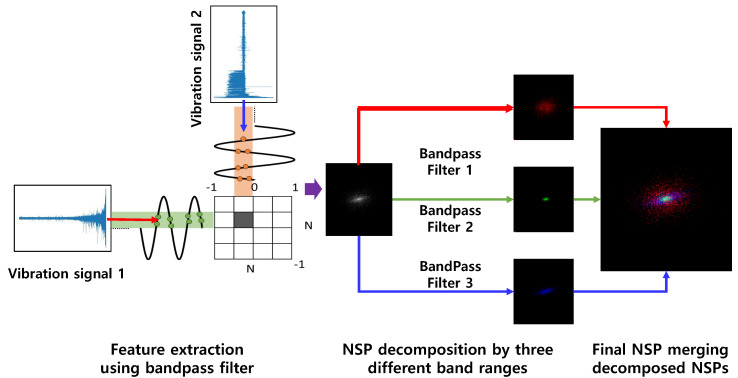
Nested-scatter plot image (NSP) methods and application to vibration signals.

By using NSP, we transform multi-channel vibration signals into a single fixed-size image. Continuous multi-channel vibration signals are split into given intervals. A three-step approach is used: feature extraction using bandpass filters, decomposition of the nested clusters in each bandwidth, and aggregation of the decomposed sections into a single RGB image. As represented in Figure 2, at least two different data channels as data sources are required and the first step is the incorporation extraction of signals in three different bandwidths. Hilbert–Huang transformation (HHT) and Fast Fourier transform (FFT) are used to determine the bandwidth of bandpass filters [25]. In the second step, the extracted two-channel signals are compressed into nested clusters. Each channel signal is mapped on the x- and y-axis. Three different extracted signals are colored in red, green, and blue, respectively. In the final stage, three scatter plots are aggregated together to form a single RGB image that is used as an input in our proposed method. An example of decomposition and merged NSP is shown in Figure 2.

### 3.2. Hs Division Prediction Using CNN

By transforming continuous raw vibration time series data into images, we can now use our data as inputs to the CNN, which has proved its outstanding performance in computer vision [26]. The structure of the proposed convolutional neural networks for the HS division model (CNN-HS) is as follows. There are three convolution (Conv) layers with kernel sizes of 10 by 10, five by five, and three by three. The Conv layers are followed by two fully connected (FC) layers. A dropout rate of 50% was applied at the end of each FC layer. The final output layer is activated by a softmax function so that the obscurity during the interim period between the two stages can be quantified by a value between zero and one. The structure is shown in Table 1.

In the CNN architecture, the selectable hyperparameters are the number of filters in each Conv layer and the size of each FC layer. In the fault detection and diagnosis using NSP [25], the optimal numbers were found by varying the number of filters in each Conv layer. The size of the FC layer affects the expressiveness of the networks and the training time. Following [25], the size of each FC layer was determined as 500 and 50. In the experiments, the number of epochs during training was 30 and the validation data ratio was 1:9. An Adam optimizer was implemented with a learning rate of 0.001.

**Figure 3:**
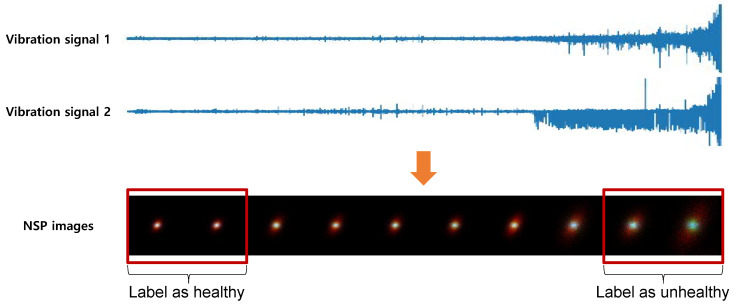
Binary labeling on part of training dataset on bearing wear experiment.

In a supervised manner, labeling of the target data is required for supervised learning to distinguish the features of the HS of a machine under different bearing degradation conditions. The training dataset is divided into two HS stages based on the time of acquisition. Figure 3 shows the division of our training dataset. The initial part, corresponding to the nominal functioning in the entire vibration dataset, is labeled as healthy, and the last part of the sample duration, when the bearing is damaged, is labeled as unhealthy. This is based on the assumption that degrading data of rotating machinery are obtained until the point of failure. By the supervised method, it is unnecessary to label all train datasets and analyze the dataset for labeling. To minimize labeling efforts on the entire dataset, the optimal sizes (small set) of the initial and last part, labeling ’healthy’ and ’unhealthy’, respectively, are evaluated (See Section 5.).

Once training is over, the performance of the trained CNN-HS is evaluated in the testing phase. The trained CNN-HS was able to classify the NSP of an external test dataset, which was not used in training, but operated under the same conditions as the rest of the training datasets. The binary regression results of the whole run-to-failure are computed, in which there is no need to specify a threshold value. As the trained CNN-HS learns the differences in the features of the NSP for healthy and unhealthy data labeled in part of the training datasets, it can recognize the degradation pattern of all of the data. For HS division, it is important to determine FPT, which is a starting point of the deterioration of bearings, in terms of maintenance. However, it is difficult to determine FPT since the features of the deterioration at FPT are weaker than the features in the unhealthy stage.

**Figure 4:**
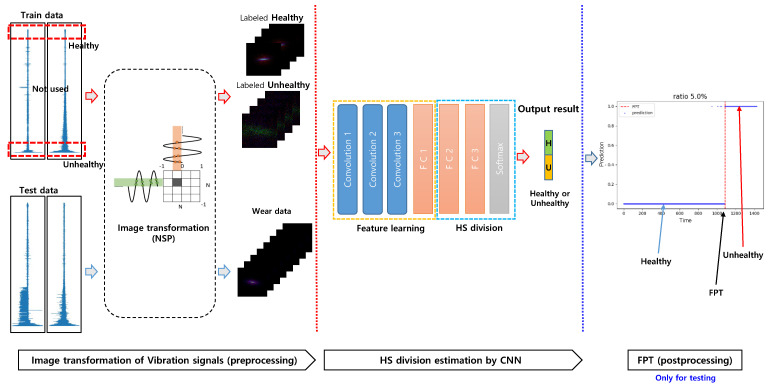
CNN-HS combined with NSP.

To determine FPT, we implemented a simple continuous trigger mechanism in which the machinery was considered to be in an unhealthy stage when a certain number of consecutive values of one (unhealthy) were given out by our model. This trigger mechanism prevents unnecessary oscillation and uncertainty to determine earlier FPT. The trigger mechanism has generally adopted an HS division strategy [2,11,27]. In the experiments, the threshold number of consecutive estimations of unhealthy is set to three based on the analysis of signals in order to prevent false alarms during the normal stages. The overview and flowchart of CNN-HS are shown in Figure 4.

## 4. Experimental Results

### 4.1. Data Description and Implementation

The nature of this study needed continuous time series data on machinery that degraded over-time to the point of rolling bearing failure, including any failures in the balls, rings, and cage. The purpose of early fault detection is to detect the degradation as early as possible so that maintenance or prognostic-based decisions can be implemented. FPT is the indicator that detects the start of the degradation. We benchmarked CNN-HS with two widely used datasets: the IMS dataset and the FEMTO dataset. Both bearing datasets were acquired from accelerated degradations of bearings in a run-to-failure manner and are widely used to demonstrate the approaches for fault diagnostics and prognostic methods.

**Figure 5:**
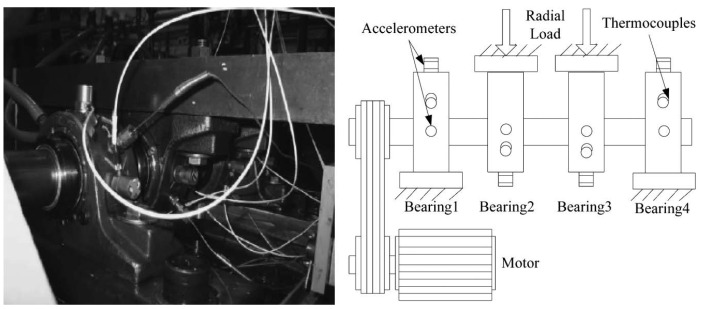
The experimental test rig and sensor placement illustration of IMS [28].

**Figure 6:**
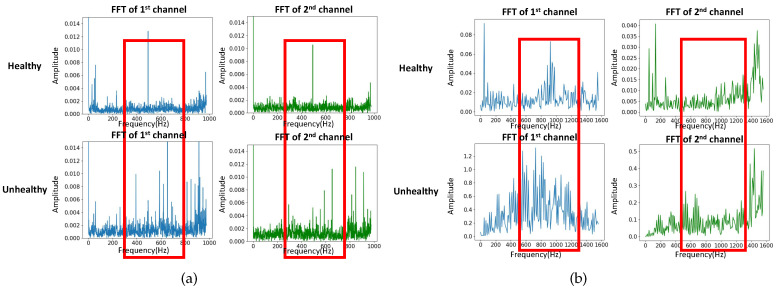
Spectral density difference in bandwidths through fast Fourier transform, (**a**) Intelligent Maintenance Systems (IMS), (**b**) Fanche-Comte Electronics Mechanics Thermal Science and Optics—Sciences and Technologies Institute (FEMTO).

The IMS dataset is generated by the NSF I/UCR Center for Intelligent Maintenance Systems (IMS) [28] and the experimental test rig and sensor placement are shown in Figure 5. The IMS dataset is composed of three sets of run-to-failure experiments in which four bearings were installed on a shaft and measured. The test was stopped when the accumulated debris exceeded a certain level and the status of each bearing was recorded. All three sets have a sampling rate of 20 kHz but differ in the data acquisition phase. Only the first set has two-channel (horizontal and vertical) bearing data and was used for our model testing and validation. We transformed the time series data into fixed-size images by using NSP. Figure 6a shows the spectral density difference in bandwidths through FFT on the IMS dataset and the comparison between healthy and unhealthy data. The spectral density from 400 to 700 Hz of unhealthy data was greater than that of healthy data. In this experiment, the sampling rate was considered by deciding the bandpass filters during the image transformation phase: 400–500 Hz, 500–600 Hz, 600–700 Hz.

Figure 3 shows the degradation process of bearing 4 of the first dataset, in which a roller element defect occurred at the end of the accelerating process. The degradation data of bearing 4 was used in our experiment to test the capability of our proposed method.

**Figure 7:**
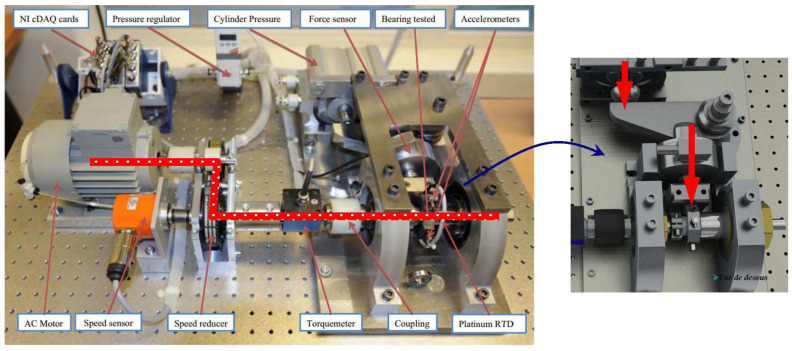
The experimental PRONOSTIA platform for accelerated bearing degradation tests [9].

The FEMTO dataset has been available to the public since the IEEE PHM 2012 Prognostic Challenge (PHM 2012). The dataset was collected on an accelerated aging platform, PRONOSTIA, as shown in Figure 7 [8,9]. Asynchronous motor, a shaft, a speed controller, and an assembly of two pulleys are used to change the speed of the rolling bearings. Similarly, the vibration data in the horizontal direction are investigated. The dataset was composed of 17 run-to-failure data in which a single bearing was tested (two columns of vibration data: horizontal and vertical). The sampling frequency of data acquisition is 25.6 kHz, which was taken into consideration in our image transformation phase. The 17 datasets are grouped into three sections by operating conditions that differ in rotation speed and radial load. Each dataset has a different run-to-failure time, requiring fault detection methods that are adaptable to time-varying operational conditions and environments. When the amplitude of the vibration data exceeds 20 g, the measurement of the run-to-failure experiments is stopped and the bearing is considered to be defective. Figure 6b shows the spectral density difference in bandwidths through FFT on the FEMTO dataset and the comparison between healthy and unhealthy data. The spectral density from 500 to 1200 Hz of unhealthy data was different from that of healthy data. Taking the sampling frequency into consideration, three different bandpass filters were used: 500–800 Hz, 800–900 Hz, 900–1200 Hz.

The NSP representation and the CNN-HS were implemented using Python scripts and the Keras library on the TensorFlow framework and were tested on a Linux system. The details of the runtime environment are shown in Table 2. The source code to reproduce our experiments is available at https://github.com/opensuh/CNNHS/.

The proposed method was compared with previously widely used methods, RMS and AE. RMS was implemented simply by calculating the square root value of the addition of the horizontal and vertical vibration data squared. The mean of the calculated values in the interval of 10 s was then used as the RMS value to compare it with other methods. The threshold was specified by testing different threshold values that produced the least false alarms, while, at the same time, making the prediction early enough before complete failure.

AE was implemented by utilizing the first 20% of the degrading data as training data. In the testing phase, the loss value calculated from the difference of the output and input of the test data was used as a metric for anomaly detection. The theory behind this was that since the first 20% of the overall data, considered healthy, was used to train the AE model, when test data that differed from the training data, which can be considered unhealthy or an anomaly, was given as an input to the AE, the difference of the output and input values would be much greater. The number of layers and nodes, the activation function, batch size, the number of epochs, and the threshold value used for fault detection were determined through different test iterations that produced the best results.

### 4.2. Evaluation of CNN-HS on IMS Dataset

**Figure 8:**
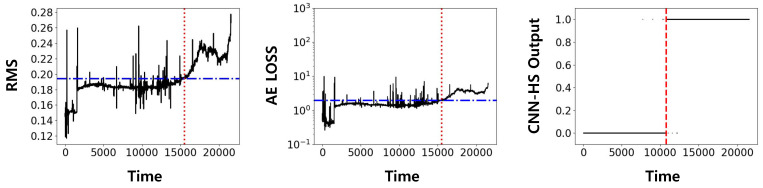
Predicted FPT using root mean square (RMS), auto-encoder (AE), and CNN-HS on IMS dataset (bearing 4). The red horizontal line is the threshold value for RMS and AE, and the red vertical line is the FPT.

In the IMS dataset, only bearing 4 in the first set was used for analysis since it showed complete failure at the end of the accelerated degradation tests. Both training and testing were done on the dataset of bearing 4 since no other bearing data in the same operational condition was available. The result of our model was then compared with previously widely used methods, RMS and AE in Figure 8. Both methods were implemented using a pre-specified threshold based on statistical properties.

Figure 8 shows the HS division in RMS, AE, and CNN-HS. RMS and AE require threshold values for HS division, and the threshold value is represented in the red horizontal line. FPT, determined by a threshold value in RMS and AE, and by the trigger mechanism in CNN-HS, is represented as a vertical red dotted line. It can be noticed that FPT given by CNN-HS is faster than that given by RMS and AE. Furthermore, the CNN-HS method has less ambiguity than other methods. Qualitatively, it can be observed that:

- HSs are clearly distinguishable from the predicted FPT.
- On the other hand, the RMS value and the loss value of AE are not stable, but show oscillations that might lead to false alarms in the case of persistent triggering.

### 4.3. Evaluation of CNN-HS on FEMTO Dataset

**Figure 9:**
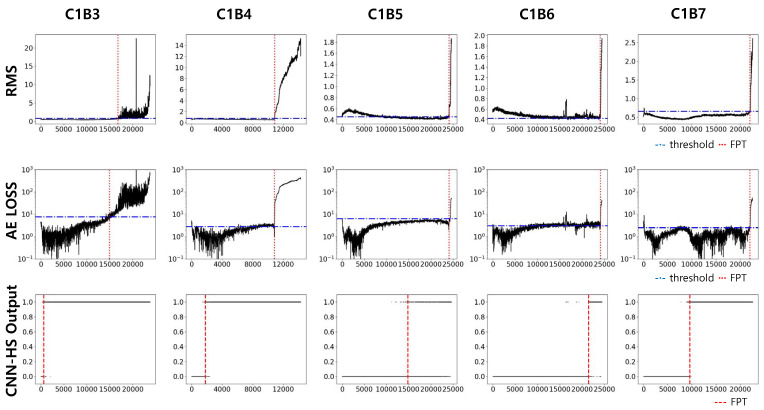
Predicted FPT using RMS, AE, and CNN-HS on FEMTO dataset (condition C1).

**Figure 10:**
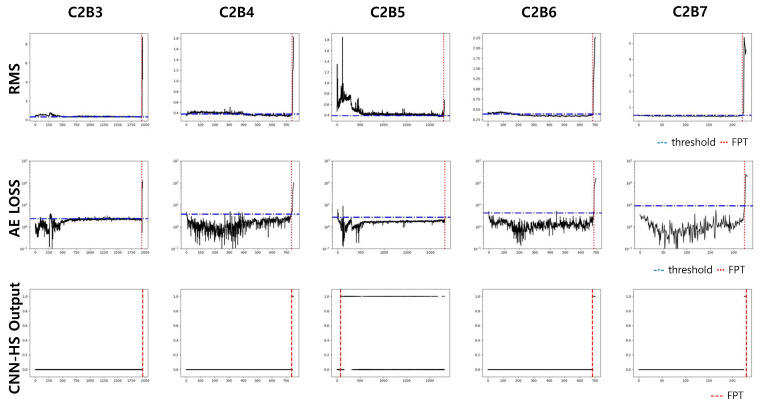
Predicted FPT using RMS, AE, and CNN-HS on FEMTO dataset (condition C2).

Datasets of bearings in condition 1 (1800 rpm and 4000 N, C1) and condition 2 (1650 rpm and 4200 N, C2) were used in our experiment. Each condition has seven run-to-failure datasets (we denote them as B1 to B7). Two datasets from each operating condition were used as the training dataset, and the trained model was tested on five other datasets in the same condition. The results of the prediction in C1 are compared with previous methods, RMS and AE, in Figure 9 and Figure 10. Again, RMS and AE require a pre-specified threshold value for HS division, and the threshold value is represented by the red horizontal line. FPT, determined by a threshold value in RMS and AE, and by a trigger mechanism in CNN-HS, is represented as a vertical red dotted line.

The FPTs measured from all the experiments in both C1 and C2 are recorded in Table 3. The test results show that our CNN-HS can detect faulty features much faster. On average, the CNN-HS method predicted faults 47.65% and 44.80% faster than the RMS and AE methods, respectively in terms of time.

**Figure 11:**
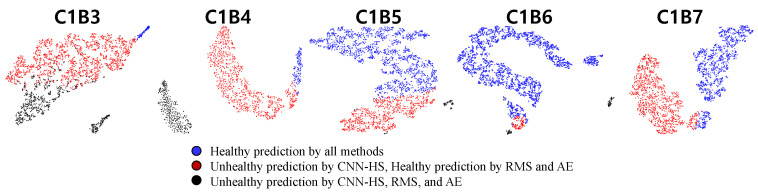
Feature maps of CNN-HS using t-Distributed Stochastic Neighbor Embedding (t-SNE) with principal component analysis (PCA) (condition C1).

**Figure 12:**
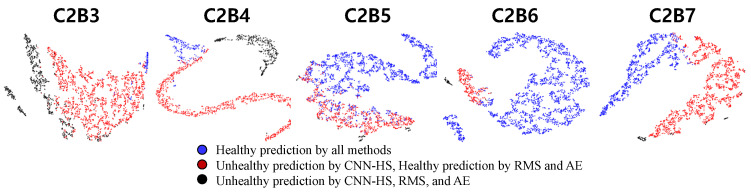
Feature maps of CNN-HS using t-SNE with PCA (condition C2).

An analysis of the features extracted by NSP and CNN-HS was conducted. We visualize features using t-Distributed Stochastic Neighbor Embedding (t-SNE) [29], which is commonly used for visualizing high-dimensional features in scatter plots and projects high-dimensional objects into two-dimensional points, such that similar objects are closer and dissimilar objects are further away from each other. The features were extracted from the output of the first fully connected (FC) layer, which consists of 500 nodes. The 500-dimensional features were then reduced to 100 dimensions by using principal component analysis (PCA) [30] and further reduced to two dimensions by using t-SNE. By this procedure, the features of NSP were mapped into a two-dimensional plane. Figure 11 and Figure 12 show the reduced features of tested bearings in C1 and C2 mapped into a two-dimensional plane.

This analysis shows that our CNN-HS model is able to detect the HS division features of NSP better than RMS and AE. Blue and black points are vibration conditions, where RMS, AE, and CNN-HS predict ’healthy’ and ’unhealthy’, respectively. The difference in FPT comes from the red points, where the CNN-HS model was able to identify the faulty features not detected by RMS- and AE-based methods. This feature map proves that the NSP is an appropriate tool for extracting useful features from raw vibration data to represent the health of machinery. The NSP was able to transform vast amounts of raw vibration signal data into a single image from which features could be extracted using the CNN-HS model. Thus, there is a clear distinction between the features that were predicted to be unhealthy and healthy by the CNN-HS method as well as compared with the predictions of other methods. Moreover, we observed that our CNN-HS method distinguishes the faulty features of the images produced by the NSP. The region of the black point is small and far away from the blue and red regions. CNN-HS combined with NSP is able to detect minor symptoms (undetected by the other two methods), which leads to more lead time for the investigation of the failure process.

## 5. Discussion

By experimental evaluation, the feasibility of our model for HS division is demonstrated. Our model is able to capture faulty features ahead of previous methods, as demonstrated in the feature maps, which generate a significant amount of lead time on the maintenance time horizon. To verify this capability, we conducted another test for the feasibility of our method for HI prediction. Thanks to the capability of the initial wearing feature extraction, our proposed method can make earlier FPT predictions than the signal-based and unsupervised data-driven method that requires specified thresholds.

In order to verify the performance of the proposed method, more experimental evaluations on the reliability of the dataset are needed. There are other benchmark datasets, but we could not work with them due to the following reasons. NSP is a scalable and signal-independent data-wrangling method, but it requires multiple channel signals. Other open benchmark datasets containing multiple channels of vibration data were not found. As of now, there are no datasets available with labeled ground truth FPT. As such, this work has been developed in the face of this existing difficulty and thus remains among the first works of this kind.

For efficient HS division using supervised learning, there is a need for an extensive amount of degradation datasets under variable operational conditions. Wearing patterns are varying in laboratory test benches as well as in real-world applications. Some wearings degrade gradually, while others degrade rapidly. A general supervised HS division method can be developed if a large amount of data covering such varying wear is made available. Presently, there is a dearth of rich data in this context. In light of this aspect, the present work becomes significantly important as it provides the early detection of FPT, exceeding the performance of common existing approaches.

More details about the selection and labeling of the training data are discussed in the following section.

### 5.1. Impact of Combination of Training Data

In our proposed method, training data from the same operating condition is needed, and the selection of the training dataset of our model plays a critical part in the performance. During our experimental phase, we tested different combinations of training data to observe how the variation in bearing degradation in the training set affected the model in both C1 and C2. The impact of the different combinations of training data in terms of the average FPT to the total duration ratio is shown in Table 4. These results show that the predicted FPT varies by up to 34.45%. The bearing datasets under accelerated degradation show two phenomena: bearing that shows gradual degradation and bearing that shows rapid degradation. From our intuition, a suspicion arises as to whether the model is predicting healthy conditions to be unhealthy prematurely since, in the training phase, relatively healthy conditions might have been labeled as unhealthy since rapid degradation was shown near the end. If this is the case, then the model might cause erroneous false alarms during healthy conditions. In the future, we plan to test our proposed method on a test bench with both degrading bearing data and normal bearing data.

### 5.2. Impact of Labeling Ratio of the Wearing Process

In the evaluation, NSP from the first 5% of the total degradation process was labeled as healthy, and the last 5% as unhealthy. This labeling makes the ratio of used training data to unused training data in the training dataset (denoted as labeling ratio) equal to 1:9. The labeling ratio of 1:9 is determined by experimental comparisons: from 1:9 to 5:5. In each condition of the FEMTO dataset, two bearing datasets are selected as training data, and the rest of the datasets are tested. First, FPT is predicted using the trained model in each test dataset. Based on the predicted FPT, the test dataset is divided into a healthy condition period (HCP) and an unhealthy condition period (UCP). The ground truth of HS division is set to ‘healthy’ for HCP data, and to ‘unhealthy’ for UCP data. The F1 score is used to evaluate the reliability of FPT made by the given labeling ratio. The F1 score is defined as follows:

(1) $$\begin{array}{c} F_{1}=\frac{2\times Recall\times Precision}{Recall+Precision} \end{array}$$

where $Recall=\frac{TP}{TP+FN}$ and $Precision=\frac{TP}{TP+FP}$. TP, FP and FN denote the true positive, false positive, and false negative values, respectively. The CNN-HS model trained with a ratio of 1:9 showed the highest average F1-score, as shown in Table 5.

### 5.3. Effectiveness of the CNN-HS Model and NSP in Representing Degradation Patterns

**Figure 13:**
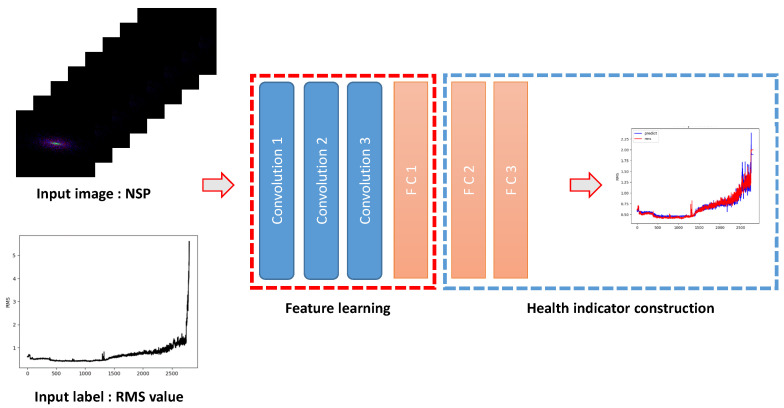
The RMS prediction model using NSP and CNN-HS.

To verify the effectiveness of the CNN-HS model and NSP in representing degradation patterns, we conducted an experiment on the FEMTO dataset. The experiment we proposed was to predict RMS values using NSP images and CNN-HS. RMS, which is a signal-based method, uses the original time series vibration data. The RMS prediction model using NSP and CNN-HS is shown in Figure 13. In the first step, the time series vibration data is transformed into NSP images and the NSP image is used as an input image of CNN-HS. In the training procedure, the RMS value of the sliding frame corresponding to the NSP image is set as an input label of CNN-HS. Unlike the proposed method, NSP images from the total degradation process are used in the training procedure. In the testing phase, the trained CNN-HS is used to compute out RMS values of NSP images acquired from the test data.

**Figure 14:**
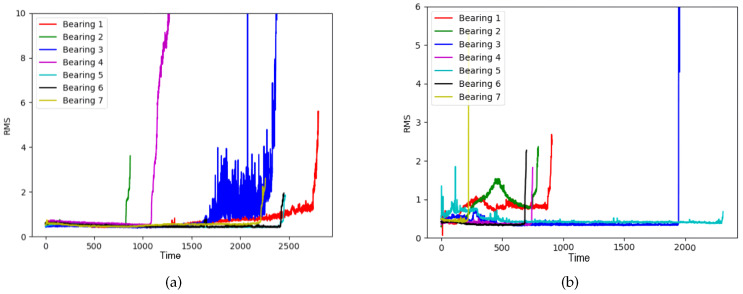
RMS values for each bearing in the FEMTO dataset, (**a**) Condition 1, (**b**) Condition 2.

**Figure 15:**
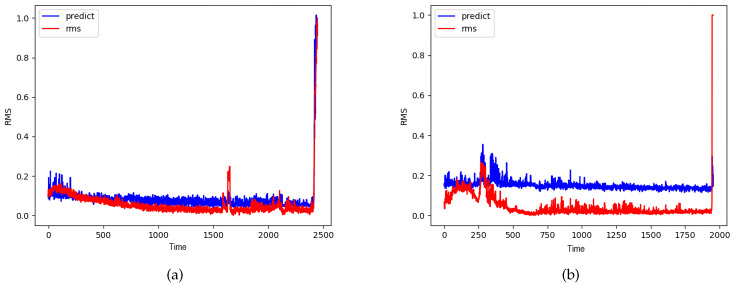
RMS value and predicted RMS value by NSP and CNN-HS in (**a**) C1B6 and (**b**) C2B3

The RMS values for each bearing under two operating conditions in the FEMTO dataset are shown in Figure 14. Because each bearing has a different degradation pattern and defect level, the pattern of RMS values and the maximum RMS value on each bearing and condition are different. To reduce the scale difference in RMS values, we saturate the maximum RMS value to a threshold value. In this experiment, we evaluated the CNN-HS with a threshold value of two because the minimum value among the maximum RMS values on bearings is two. In order to verify the accuracy of the RMS prediction using NSP and CNN-HS, the root mean square error (RMSE) is used to calculate the accuracy of the prediction values. The RMSE is defined as follows.

(2) $$\begin{array}{c} RMSE=\sqrt{\frac{1}{n}\sum_{t=1}^{n}{\left(RMS_{t}-P_{t}\right)}^{2}} \end{array}$$

where $RMS_{t}$ is the RMS value at time *t* and $P_{t}$ is the predicted RMS value by the CNN-HS model. The RMSEs are shown in Table 6. The RMSE of C1B3 is higher than the RMSE of other bearings since the RMS value of C1B3 oscillates greatly. Despite the high RMSE of C1B3, most of the RMSE values are quite low. Figure 15 shows that the predicted RMS value by CNN-HS model has very similar patterns to the RMS values. Therefore, NSP can represent the features of time series vibration data and CNN-HS can accurately predict the health stage of rolling bearings.

## 6. Conclusions

In this paper, we propose a novel method of supervised health stage prediction using CNN for bearing wear. In signal-based and unsupervised data-driven methods, threshold values have to be manually specified, a method which is prone to either high false alarm rates or low recall. The combination of NSP, which allows us to integrate a frequency analysis that helps to extract faulty wear features, and a CNN-based binary regression model, rids the necessity of designating a threshold, one of the most crucial problems in early fault detection. Our CNN-HS model effectively distinguishes healthy and unhealthy states in an unambiguous manner by changing the anomaly detection problem into a binary regression problem, utilizing a simple trigger mechanism. In order to minimize the labeling process in supervised learning, a small portion of the dataset is utilized for training the model. The additional experiments showed that the prediction error in terms of the RMSE of the RMS value prediction was quite low.

Moreover, the experimental results show that the combination of these two methods detects faults earlier than previous methods. The FPT in the experimental results showed that our CNN-HS model predicted faults 47.65% and 44.80% faster than the RMS and AE methods, respectively. Overall, we can conclude that NSP captures the feature of raw time series data efficiently, thus allowing for earlier fault predictions, and that CNN-HS predicts the health stage of bearing wear accurately, ridding us of the need for manual threshold specification.

Despite the fast and accurate health stage prediction results achieved by the proposed method, it does require a sufficient amount of training data for model development. In real industrial scenarios, supervised data with degradation is difficult to collect. In future research, we plan to divide the health state into numerous stages based on degradation time instead of just healthy and unhealthy in a supervised manner. This model could act as a health indicator and be used to recognize patterns that could be used for RUL prediction. Moreover, we plan to extend the use of NSP images in an unsupervised manner for true condition-independent fault detection methods.

## Figures and Tables

**Table 1:** The structure of the convolutional neural network (CNN)-health stage (HS).

Layers	Activation Function	Dimension
Input	-	128 × 128 × 3
Conv 10 × 10	ReLU	60 × 60 × 20
Conv 5 × 5	ReLU	28 × 28 × 40
Conv 3 × 3	ReLU	24 × 24 × 20
Fully Connected	ReLU	500
Fully Connected	ReLU	50
Output	Softmax	2

**Table 2:** Runtime environment details.

Category	Specification
CPU	Intel Core i7-7700K
GPU	NVIDIA Tesla V100-DGXS 32GB
OS	Ubuntu 16.04 LTS (Linux)
SW libraries	Python 3.7.6/CUDA v10.0.130/
	Tensorflow 2.0.0

**Table 3:** Predicted FPT in FEMTO dataset (condition C1 and C2, unit: seconds).

Bearing (End Time)	RMS	AE	CNN-HS
C1B3 (23,750)	16,760	14,940	740
C1B4 (14,280)	10,870	10,850	1880
C1B5 (24,630)	24,100	24,130	16,670
C1B6 (24,480)	24,070	24,130	21,590
C1B7 (22,590)	22,010	21,990	8800
C2B3 (19,950)	19,390	19,390	19,950
C2B4 (7510)	7400	7350	7430
C2B5 (23,110)	22,920	23,110	930
C2B6 (7010)	6840	6850	6870
C2B7 (2300)	2210	2230	2250

**Table 4:** FPT to total duration ratio in different training data variation in CNN-HS under conditions 1 and 2.

Bearing (Test)	Bearing (Train)		
	C1-B1B2	C1-B1B4	C1-B3B4
C1B5	67.6%	79.2%	87.0%
C1B6	88.1%	86.9%	97.0%
C1B7	38.9%	51.2%	60.0%
	**C2-B3B4**	**C2-B1B3**	**C2-B1B2**
C2B5	2.9%	2.4%	4.0%
C2B6	5.3%	11.8%	98.0%
C2B7	2.0%	5.6%	97.8%

**Table 5:** The reliability (F1 score) of the predicted FPT corresponding to the labeling ratio.

Condition	Labeling Ratio				
	1:9	2:8	3:7	4:6	5:5
C1	0.91	0.89	0.87	0.86	0.87
C2	0.97	0.95	0.82	0.77	0.83
C1 + C2	0.94	0.92	0.85	0.82	0.85

**Table 6:** The prediction errors using CNN-HS combined with NSP.

Bearing	RMSE	Bearing	RMSE
C1B3	0.4920	C2B3	0.1522
C1B4	0.2010	C2B4	0.0962
C1B5	0.1028	C2B5	0.1097
C1B6	0.0774	C2B6	0.1503
C1B7	0.2156	C2B7	0.1645
Average	0.2529	Average	0.1271

## Abbreviations

The following abbreviations are used in this manuscript:

AE	Auto-Encoder
CNN	Convolutional Neural Networks
CNN-HS	CNN for HS division model
FC	Fully Connected
FEMTO	Fanche-Comte Electronics Mechanics Thermal science and Optics-sciences and technologies institute
FFT	Fourier Transform
FPT	First Predicting Time
HCP	Healthy Condition Period
HHT	Hilbert Huang Transformation
HI	Health Indicator
HS	Health Stage
IMS	the center of Intelligent Maintenance System
NSP	Nested-Scatter Plot
PCA	Principal Component Analysis
RMS	Root Mean Square
RUL	Remaining Useful Life
t-SNE	t-distributed Stochastic Neighbor Embedding
UCP	Unhealthy Condition Period

## References

[1] Liang X., Zuo M.J., Feng Z. (2018). Dynamic modeling of gearbox faults: A review. Mech. Syst. Signal Process..

[2] Lei Y., Li N., Guo L., Li N., Yan T., Lin J. (2018). Machinery health prognostics: A systematic review from data acquisition to RUL prediction. Mech. Syst. Signal Process..

[3] Atamuradov V., Medjaher K., Dersin P., Lamoureux B., Zerhouni N. (2017). Prognostics and health management for maintenance practitioners-Review, implementation and tools evaluation. Int. J. Progn. Health Manag..

[4] Wang W. (2002). A model to predict the residual life of rolling element bearings given monitored condition information to date. IMA J. Manag. Math..

[5] Fan J., Wang W., Zhang H. AutoEncoder based high-dimensional data fault detection system. Proceedings of the 2017 IEEE 15th International Conference on Industrial Informatics (INDIN).

[6] Principi E., Rossetti D., Squartini S., Piazza F. (2019). Unsupervised electric motor fault detection by using deep autoencoders. IEEE/CAA J. Autom. Sin..

[7] Qiu H., Lee J., Lin J., Yu G. (2006). Wavelet filter-based weak signature detection method and its application on rolling element bearing prognostics. J. Sound Vib..

[8] Nectoux P., Gouriveau R., Medjaher K., Ramasso E., Chebel-Morello B., Zerhouni N., Varnier C. PRONOSTIA: An experimental platform for bearings accelerated degradation tests. Proceedings of the IEEE International Conference on Prognostics and Health Management (PHM’12).

[9] Javed K., Gouriveau R., Zerhouni N., Nectoux P. A feature extraction procedure based on trigonometric functions and cumulative descriptors to enhance prognostics modeling. Proceedings of the 2013 IEEE Conference on Prognostics and Health Management (PHM).

[10] Paris P., Erdogan F. (1963). A critical analysis of crack propagation laws. J. Basic Eng..

[11] Li N., Lei Y., Lin J., Ding S.X. (2015). An improved exponential model for predicting remaining useful life of rolling element bearings. IEEE Trans. Ind. Electron..

[12] Randall R.B., Antoni J. (2011). Rolling element bearing diagnostics—A tutorial. Mech. Syst. Signal Process..

[13] Gao Z., Cecati C., Ding S.X. (2015). A survey of fault diagnosis and fault-tolerant techniques—Part I: Fault diagnosis with model-based and signal-based approaches. IEEE Trans. Ind. Electron..

[14] Bouzid M.B.K., Champenois G. (2012). New expressions of symmetrical components of the induction motor under stator faults. IEEE Trans. Ind. Electron..

[15] Wang Y., Peng Y., Zi Y., Jin X., Tsui K.L. (2016). A two-stage data-driven-based prognostic approach for bearing degradation problem. IEEE Trans. Ind. Inform..

[16] Qian Y., Yan R., Hu S. (2014). Bearing degradation evaluation using recurrence quantification analysis and Kalman filter. IEEE Trans. Instrum. Meas..

[17] Jha M.S., Dauphin-Tanguy G., Ould-Bouamama B. (2016). Particle filter based hybrid prognostics for health monitoring of uncertain systems in bond graph framework. Mech. Syst. Signal Process..

[18] Wen L., Li X., Gao L., Zhang Y. (2017). A new convolutional neural network-based data-driven fault diagnosis method. IEEE Trans. Ind. Electron..

[19] Razavi-Far R., Farajzadeh-Zanjani M., Zare S., Saif M., Zarei J. One-class classifiers for detecting faults in induction motors. Proceedings of the 2017 IEEE 30th Canadian Conference on Electrical and Computer Engineering (CCECE).

[20] Belmiloud D., Benkedjouh T., Lachi M., Laggoun A., Dron J. (2018). Deep convolutional neural networks for Bearings failure predictionand temperature correlation. J. Vibroeng..

[21] Guo L., Lei Y., Li N., Yan T., Li N. (2018). Machinery health indicator construction based on convolutional neural networks considering trend burr. Neurocomputing.

[22] She D., Jia M. (2019). Wear indicator construction of rolling bearings based on multi-channel deep convolutional neural network with exponentially decaying learning rate. Measurement.

[23] Li X., Zhang W., Ding Q. (2019). Deep learning-based remaining useful life estimation of bearings using multi-scale feature extraction. Reliab. Eng. Syst. Saf..

[24] Jo J., Lee Y.O., Hwang J. Multi-Layer Nested Scatter Plot a Data Wrangling Method for Correlated Multi-Channel Time Series Signals. Proceedings of the 2018 First International Conference on Artificial Intelligence for Industries (AI4I).

[25] Suh S., Lee H., Jo J., Lukowicz P., Lee Y.O. (2019). Generative Oversampling Method for Imbalanced Data on Bearing Fault Detection and Diagnosis. Appl. Sci..

[26] Rawat W., Wang Z. (2017). Deep convolutional neural networks for image classification: A comprehensive review. Neural Comput..

[27] Wang P., Wang H., Yan R. (2019). Bearing degradation evaluation using improved cross recurrence quantification analysis and nonlinear auto-regressive neural network. IEEE Access.

[28] Yu J. (2012). Health condition monitoring of machines based on hidden Markov model and contribution analysis. IEEE Trans. Instrum. Meas..

[29] Maaten L.v.d., Hinton G. (2008). Visualizing data using t-SNE. J. Mach. Learn. Res..

[30] Wold S., Esbensen K., Geladi P. (1987). Principal component analysis. Chemom. Intell. Lab. Syst..

